# Anxiety and its associated factors among inmates in ARBA Minch and JINKA town, southern Ethiopia

**DOI:** 10.1186/s12888-022-04230-5

**Published:** 2022-09-01

**Authors:** Hanan Abdulkadir, Meseret Girma, Zeleke Gebru, Negussie Boti Sidamo, Gebremaryam Temesgen, Kiyar Jemal

**Affiliations:** 1 School of Public Health, College of Medicine and Health Sciences, Arba Minch University, Arba Minch, Ethiopia; 2 Department of Midwifery, College of Medicine and Health Sciences, Arba Minch University, Arba Minch, Ethiopia; 3 Departement of laboratory, College of Medicine and Health Sciences, Arba Minch University, Arba Minch, Ethiopia

**Keywords:** Anxiety, Prisoners, Ethiopia

## Abstract

**Background:**

The prison populations are more affected by mental illness than the general population but little attention is given to this population. And there is a limitation of study on the magnitude of anxiety and its associated factors. So this study aimed to assess the magnitude of anxiety and its associated factors among prisoners in Arba Minch and Jinka town, Ethiopia.

**Methods:**

An institution-based cross-sectional study was conducted among 650 randomly selected prisoners at Arba Minch and Jinka town. Data was collected by using open data kit then converted to excel and exported to SPSS version 25 for analysis. Descriptive statistics including frequency, means, and proportion were performed. Binary logistic regression was used to identify the associated variables. *P* < 0.05 was used to declare statistical significance.

**Results:**

The proportion of Anxiety among prisoners was 28% [*N* = 174, (95%CI, 25–32%)]. Age ≥ 48 (AOR = 4.21, 95%CI 1.99–8.93), age 38–47 (3.95, 1.94–8.07), being an urban resident (2.48, 1.56–3.95), not doing physical exercise (2.71, 1.53–4.79), having a chronic disease (1.63, 1.07–2.47), having 1–2 stressful life events (2.92, 1.59–5.35), duration of punishment > 5 years (2.92, 1.59–5.35) and lack of income-generating job in prison (2.54, 1.48–4.35) were significantly associated with anxiety.

**Conclusion:**

The magnitude of anxiety among prisoners was high. Age ≥ 48 and 38–47, urban residence, not doing physical exercise, having a chronic disease, having 1–2 stressful life events, duration of punishment > 5 years, and lack of income-generating job in prison were significantly associated with anxiety. Giving special attention and being supportive to older age and those who have a chronic disease, facilitating physical exercise, creating job opportunity in the prison, and giving training for prisoners on stress coping mechanism and anxiety is recommended.

## Background

Anxiety is a mental disease marked by restlessness and a sense of being “on edge,” uncontrollable feelings of concern, increased irritability, concentration problems, and sleep problems, such as difficulty sleeping or staying asleep [1].

Anxiety disorders affect 3.6% of people globally, or around 264 million people, according to the World Health Organization. Additionally, anxiety affects 4.6% of females and 2.6% of males worldwide [2]. Mental health issues appear to be becoming more prevalent in Africa. As a result of mental health issues, 17.9 million years were lost to incapacity in 2015 [3]. Worldwide there are more than 10.35 million people in penal institutions. Since the year 2000, the world prison population total has grown by almost 20% [4]. The prevalence of the mental disorder is higher among prisoners than in the general population [5]. Previous studies showed that the magnitude of anxiety among prisoners ranges from 77.8–8% [6, 7]. A study by the Ministry of Justice found that 23% of male and 49% of female prisoners in Australia suffer from depression and anxiety [8]. The study done in North West Ethiopia showed that the prevalence of anxiety among prisoners was 36.1% [9].

Anxiety can cause a significant problem in social interaction, school, and work. It also has long-term effects like increased risk of heart disease, lowered immune system, irritable bowel syndrome, memory problems, and frequent migraines [10, 11]. Studies showed that Age, marital status, self-reported physical and mental health, previous mental illness, imprisonment status, prison accommodation, prison meal, health care services, unhappy life before imprisonment, smoking, and residence were associated with anxiety among prisoners [6, 9, 12].

Mental disorder among prisoners is a public health problem. However, there is limited information related to the magnitude of anxiety and its associated factors among prisoners in Ethiopia, especially in the study area. So this study aims to assess the magnitude of anxiety and its associated factors among prisoners in the correctional institutions of Arba Minch and Jinka town, Ethiopia.

## Methods

### Study setting and design

An institution-based cross-sectional study was conducted from July 04 up to 18, 2021in the prisons of Arba Minch and Jinka town, Ethiopia. Arba Minch Town, the capital of the Gamo Zone, is 275 km from the regional town of Hawassa and 450 km from Addis Ababa. Jinka Town, the South Omo zone’s center, is 520 km from the regional town of Hawassa and 750 km from Addis Ababa. The Arba Minch Town Correctional Institution was founded in 1955E.C. and it now houses 1156 inmates. The Jinka Town Correctional Institution was founded in 1967 E.C. and now houses 760 inmates.

### Sample size determination and sampling procedure

The study population consisted of all prisoners housed at Arba Minch and Jinka Town prisons. The study excluded prisoners who were openly imprisoned, seriously ill during data collection, and those who were awaiting trial. A total of 650 sample size was computed by the single population proportion formula by assuming a 4% margin of error, 95% confidence interval, and 56.4% proportion of depression among prisoners in Hawassa prison, Southern Ethiopia [12] and 10% non-response rate. All inmates in Arba Minch and Jinka town were included in this study. The proportional to size allocation was applied for each prison after the total numbers of prisoners were identified in each prison. Also, the female and the male prisoners were proportionally allocated according to their size. Study participants were selected by simple random sampling from the sampling frame obtained from registration books at the prison.

### Study variables

#### Dependent variable

Anxiety.

#### Independent variables

- **Socio-demographic related variables:** Age, sex, marital status**,** residence**,** educational status, occupational status, and having children.
- **Behavioral-related variables:** Current substance use such as alcohol use, cigarette smoking, khat chewing and using smokeless tobacco, and physical exercise.
- **Stressful life event and clinical disease-related variables:** Stressful life event, history of chronic illness, and family history of mental illness.
- **Prison situation-related variables:** Social support, history of previous incarceration, lack of job in prison, duration of imprisonment, and duration of punishment.

### Operational definitions

#### Anxiety

For measuring anxiety, the Generalized Anxiety Disorder 7-item (GAD-7) scale was used with high sensitivity of 89% and a specificity of 82%. An individual with a score of 10 or more was considered to have anxiety which was the obtained cutoff value [9, 13].

#### Level of social support

For this study social support was measured using the Oslo 3-item social support scale. The sum score ranges from 3 to 14, with high values representing strong levels and low values representing poor levels of social support. The OSSS-3 sum score can be operationalized into three broad categories of social support with a score of 3–8 indicating poor support, 9–11 moderate support, and 12–14 strong support [14, 15].

#### Presence of stressful life events

Refers to Individuals who had at least one or more stressful life events (close family member died, divorce, serious illness or injury in the family member, etc.…..) in the last four weeks. The LTE contains 12 categories of significant life events, for example relating to the death of close persons, loss of relationships, imprisonment, and being the victim of theft. These 12 categories accounted for two-thirds of all events collected in the original development of the tool. Thus the experience of life events was grouped into three categories (none; 1–2 life events and 3 and above) [16].

#### Chronic illness

Having one disease like diabetes, hypertension, eye problem, heart disease, HIV, or kidney disease. Reported from the patient that is confirmed by the health professional.

### Data collection tool and quality control

Pretested structured interviewer-administered questionnaire which had five parts was used for data collection. These were socio-demographic, behavioral factors, clinical disease-related factors, prison situation-related factors, and GAD-7. Each question on GAD-7 measures a problem that the prisoners were bothered by in the last 15 days. Anxiety symptom was rated on a scale ranging from zero (not at all) to three (nearly every day). Anxiety total scores were computed for every one of the participants by adding scores of all the seven items of the scale. A participant was considered to be in a state of depression if he/she scored ten and above [7, 13]. The stressful life event will be assessed by using an adapted version of the List of threatening experiences (LTE) questionnaire. It is a twelve items instrument measuring common life events that tend to be threatening. This instrument has been adapted and used previously in the Ethiopian setting [16, 17]. The behavioral-related questionnaire was adapted from the WHO Stepwise approach. Behavioral related focused on substance use and history of leisure-time physical exercise. Finally, part of the questionnaire asked about prison situation-related questions like (previous incarceration, duration of imprisonment, duration of punishment, and income generating jobs in prison) and social support. The tool was first developed in the English language and translated to the Amharic language then back to the English language to check the consistency. Ten (10) data collectors and six (6) supervisors (five data collectors and three supervisors for each correctional institution) who were health professionals who had direct experience and the ability to speak the Amharic language were recruited for data collection. Data quality was ensured during instrument development, collection, coding, and analysis. The supervisors and data collectors were trained about the purpose of the study and how to supervise and collect questionnaires respectively. The tool was pre-tested on 5% of the study participants in a correctional institution of Sawla correctional institution (which is not included in this study), before the actual data collection. During data collection, the questionnaire was checked for its completeness daily by supervisors and then by investigators. Intensive supervision was carried out by the assigned trained supervisors and research team members throughout the data collection period. This helped to identify problems that were addressed in the questionnaires.

### Data processing and analysis

Data was collected using ODK and converted to excel then exported to SPSS version 25 for analysis. The frequency distribution of socio-demographic factors was studied after data cleaning and modification. Then, for each independent variable and outcome of interest, a binary logistic regression analysis was used to find independent factors linked to the outcome. After the binary logistic regression analysis was completed, variables with a *p*-value of less than 0.25 were chosen for the multiple logistic regression analysis. Context and previous studies were also taken into account while selecting a variable for multivariable analysis. The p-value associated with each parameter was evaluated with a cut-off point of less than 0.05 to determine whether or not a variable is significant. The crude and adjusted odds ratios, as well as their 95% confidence intervals, were calculated and interpreted as needed. Hosmer and Lemeshow tests were used to verify the final model’s goodness of fit.

## Result

### Socio-demographic characteristics

From the total sample size, 618 participants were volunteers making the response rate 95.08%. Of the age groups, more than one-third 60 (34.5%) of the prisoners who had anxiety were aged 18–27. Of the sex groups, about 124 (71.3%) of inmates who had anxiety were male. Regarding marital status more than half 103 (59.2%) of the inmates who had anxiety were those who were married. Related to a residence more than half 98 (56.3%) of the participants who had anxiety were urban residents. Of the occupations, about 128 (73.6%) who had anxiety were employed. About 126 (72.4%) of inmates who have a child had anxiety (Table 1).

**Table 1:** Socio-demographic characteristic of prisoners in Arba Minch and Jinka town, Southern Ethiopia, 2021

Variables (*n* = 618)	Categories	Anxiety	
		Yes	No
Age (years)	18–27	60 (34.5%)	241 (54.3%)
	28–37	41 (23.6%)	118 (26.6%)
	38–47	39 (22.4%)	42 (9.5%)
	≥48	34 (19.5%)	43 (9.7%)
Sex	Male	124 (71.3%)	376 (84.7%)
	Female	50 (28.7%)	68 (15.3%)
Marital status	Married	103 (59.2%)	272 (61.3%)
	Single	46 (26.4%)	153 (34.5%)
	Divorced or widowed	25 (14.4%)	19 (4.3%)
Residence	Urban	98 (56.3%)	189 (42.6%)
	Rural	76 (43.7%)	255 (57.4%)
Educational status	No formal education	34 (19.5%)	77 (17.3%)
	Elementary	72 (41.4%)	219 (49.3%)
	High school	58 (33.3%)	123 (27.7%)
	Collage and above	10 (5.7%)	25 (5.6%)
Occupation	Employed	128 (73.6%)	367 (82.7%)
	House wife	12 (6.9%)	9 (2.0%)
	Student	10 (5.7%)	41 (9.2%)
	Un-employed	24 (13.8%)	27 (6.1%)
Have children	Yes	126 (72.4%)	273 (61.5%)
	No	48 (27.6%)	171 (38.5%)

### Behavioral characteristics

Related to using smokeless tobacco less than one-third 25 (14.4%) of the prisoners who had anxiety were those who use smokeless tobacco. Regarding physical exercise majority, 142 (81.6%) of the inmates who had anxiety were those who didn’t do physical exercise (Table 2).

**Table 2:** Behavioral characteristic of prisoners in Arba Minch and Jinka town, Southern Ethiopia, 2021

Variable (n = 618)	Categories	Anxiety	
		Yes	No
Using smokeless tobacco	Yes	25 (14.4%)	69 (15.5%)
	No	149 (85.6%)	375 (84.5%)
Physical exercise	Yes	32 (18.4%)	203 (45.7%)
	No	142 (81.6%)	241 (54.3%)

### Clinical characteristics, stressful life events, and social support

More than half 99 (56.9%) of the participants who had anxiety were those who had chronic diseases. Less than one-third 20 (11.5%) of the prisoners who had family mental illness had anxiety. About 42 (24.1%) of the participants who had 3 and above stressful life events had anxiety**.** Majority148 (85.1%) of the inmates who had anxiety were those who had poor social support (Table 3).

**Table 3:** Clinical characteristics, stressful life event and social support of prisoners in Arba Minch and Jinka town, Southern Ethiopia, 2021

Variables (*n* = 618)	Categories	Anxiety	
		Yes	No
Chronic disease	Yes	99 (56.9%)	180 (40.5%)
	No	75 (43.1%)	264 (59.5%)
Family history of mental illness	Yes	20 (11.5%)	47 (10.6%)
	No	154 (88.5%)	397 (89.4%)
Stressful life event	None	98 (56.3%)	225 (50.7%)
	1–2	34 (19.5%)	147 (33.1%)
	3 and above	42 (24.1%)	72(16.2%)
Social support	Poor	148 (85.1%)	320 (72.1%)
	Moderate	17 (9.8%)	77 (17.3%)
	Strong	9 (5.2%)	47(10.6%)

### Prison situation

The majority 145 (83.3%) of the inmates who had anxiety were those whose duration of punishment was> 5 years. About 146 (83.9%) of the prisoners who had anxiety were those who didn’t have an income-generating job in prison (Table 4).

**Table 4:** Prison situation of prisoners in Arba Minch and Jinka town, Southern Ethiopia, 2021

Variables (*n* = 618)	Categories	Anxiety	
		Yes	No
Duration of punishment (in year)	< 1 year	1 (0.6%)	12 (2.7%)
	1–5 year	28 (16.1%)	134 (30.2%)
	> 5 year	145 (83.3%)	298 (67.1%)
Duration of imprisonment (in year)	< 1 year	37 (21.3%)	132 (29.7%)
	1–5 year	65 (37.4%)	183 (41.2%)
	> 5 year	72 (41.4%)	129 (29.1%)
History of previous incineration	Yes	15 (8.6%)	30 (6.8%)
	No	159 (91.4%)	414 (93.2%)
Income generating job in prison	Yes	28 (16.1%)	157 (35.4%)
	No	146 (83.9%)	287 (64.6%)

### Magnitude of anxiety

The proportion of anxiety among prisoners was 28.2% [*N* = 174 (95%CI, 25–32%)]. Less than one-third 78 (12.6%) of the prisoners had a nervous feelings and were anxious every day. Whereas about 147 (23.8%) of respondents were not able to stop or control worrying for several days. Less than one-third 81 (13.1%) of the participants had trouble relaxing for nearly half the days. About 152 (24.6%) of the prisoners had a feeling of being easily annoyed or irritable for several days (Table 5).

**Table 5:** Magnitude of anxiety on prisoners in the correctional institutions of Arba Minch and Jinka town, Southern Ethiopia, 2021

Variables (*n* = 618)	Categories n (%)			
	Not at all	Several days	Nearly half the days	Every day
Feeling nervous, anxious or on edge	239 (38.7)	166 (26.9)	135 (21.8)	78 (12.6)
Not being able to stop or control worrying	292 (47.2)	147 (23.8)	99 (16.0)	80 (12.9)
Worrying too much about different things	265 (42.9)	157 (25.4)	116 (18.8)	80 (12.9)
Trouble relaxing	291 (47.1)	152 (24.6)	81 (13.1)	94 (15.2)
Being so restless that it is hard to sit still	340 (55.0)	129 (20.9)	68 (11.0)	81 (13.1)
Becoming easily annoyed or irritable	228 (36.9)	152 (24.6)	139 (22.5)	99 (16.0)
Feeling afraid as if something awful might happen	287 (46.4)	132 (21.4)	103 (16.7)	96 (15.5)

### Factors associated with anxiety

Variables like age, sex, marital status, occupation, residence, having a child, physical exercise, chronic disease, social support, stressful life event, duration of punishment, duration of imprisonment, and income-generating job in prison were associated with anxiety in bivariate association and were a candidate for the multivariable association. Then age, residence, physical exercise, chronic disease, stressful life events, duration of punishment, and income-generating job in prison were significantly associated with anxiety.

The odds of developing anxiety were 4.21 (95%CI 1.99–8.93) and 3.95 (95%CI 1.94–8.07) times higher among prisoners whose age was ≥48 and 38–47 respectively than those aged 18–27. Those prisoners who lived in urban were 2.48 (95%CI 1.56–3.95) times more likely to develop anxiety than rural residents. The odds of developing anxiety were 2.71 (95%CI 1.53–4.79) times higher among inmates who didn’t do physical exercises than those who did physical exercise. The odds of developing anxiety were 1.63 (95%CI 1.07–2.47) times higher among prisoners who had chronic diseases than those who didn’t have a chronic disease. Inmates who had 1–2 stressful life events were 2.92 (95%CI 1.59–5.35) than those who hadn’t stressful life events. Those inmates whose duration of punishment was > 5 years were 2.92 (95%CI 1.59–5.35) times more likely to develop anxiety than those whose punishment duration is < 1 year. The odds of developing anxiety were 2.54 (95%CI 1.48–4.35) times higher among prisoners who hadn’t income-generating jobs in prison than those who had income-generating jobs (Table 6).

**Table 6:** Factors associated with anxiety among prisoners in the correctional institutions of Arba Minch and Jinka town, Southern Ethiopia, 2021

Variable	Categories	Anxiety		OR(95% confidence interval)		*P-Value*
		Yes	No	COR (95%CI)	AOR (95%CI)	
Age	18–27	60 (34.5%)	241 (54.3%)	1	1	
	28–37	41 (23.6%)	118 (26.6%)	1.396 (0.886–2.198)	1.527 (0.854–2.730)	0.153
	38–47	39 (22.4%)	42 (9.5%)	3.730 (2.218–6.271)	3.955 (1.939–8.066)	0.001*
	≥48	34 (19.5%)	43 (9.7%)	3.176 (1.867–5.403)	4.215 (1.990–8.929)	0.001*
Sex	Male	124 (71.3%)	376 (84.7%)	1	1	
	Female	50 (28.7%)	68 (15.3%)	2.230 (1.468–3.386)	1.686 (0.955–2.976)	0.072
Marital status	Married	103 (59.2%)	272 (61.3%)	1	1	
	Single	46 (26.4%)	153 (34.5%)	0.794 (0.532–1.185)	1.531 (0.714–3.280)	0.273
	Divorce or widowed	25 (14.4%)	19 (4.3%)	3.475(1.836–6.578)	2.112 (0.980–4.548)	0.056
Occupation	Employed	128 (73.6%)	367 (82.7%)	1	1	
	House wife	12 (6.9%)	9 (2.0%)	3.823 (1.574–9.285)	2.606 (0.942–7.209)	0.065
	Student	10 (5.7%)	41 (9.2%)	0.699 (0.340–1.437)	0.982 (0.409–2.357)	0.967
	Un-employed	24 (13.8%)	27 (6.1%)	2.549 (1.419–4.577)	2.006 (0.993–4.052)	0.052
Residence	Urban	98 (56.3%)	189 (42.6%)	1.740 (1.221–2.478)	2.479 (1.556–3.948)	0.001*
	Rural	76 (43.7%)	255 (57.4%)	1	1	
Have children	Yes	126 (72.4%)	273 (61.5%)	0.608 (0.414–0.892)	1.136 (0.546–2.362)	0.733
	No	48 (27.6%)	171 (38.5%)	1	1	
Physical exercise	Yes	32 (18.4%)	203 (45.7%)	1	1	
	No	142 (81.6%)	241 (54.3%)	3.738 (2.440–5.726)	2.710 (1.535–4.786)	0.001*
Chronic disease	Yes	99 (56.9%)	180 (40.5%)	1.936 (1.358–2.761)	1.629 (1.074–2.471)	0.022*
	No	75 (43.1%)	264 (59.5%)	1	1	
Stressful life event	None	98 (56.3%)	225 (50.7%)	1	1	
	1–2	34 (19.5%)	147 (33.1%)	0.531(0.341–0.826)	1.112 (0.642–1.927)	0.704
	3 and above	42 (24.1%)	72(16.2%)	1.339 (0.855–2.097)	2.918 (1.591–5.353)	0.001*
Social support	Poor	148 (85.1%)	320 (72.1%)	2.415 (1.153–5.059)	1.137 (0.479–2.696)	0.771
	Moderate	17 (9.8%)	77 (17.3%)	1.153 (0.476–2.795)	0.944(0.350–2.547)	0.909
	Strong	9 (5.2%)	47(10.6%)	1	1	
Duration of punishment (in year)	< 1 year	1 (0.6%)	12 (2.7%)	1	1	
	1–5 year	28 (16.1%)	134 (30.2%)	2.507 (0.313–20.076)	5.726 (0.552–59.372)	0.144
	> 5 year	145 (83.3%)	298 (67.1%)	5.839 (0.752–45.339)	2.918 (1.591–5.353)	0.022*
Duration of imprisonment (in year)	< 1 year	37 (21.3%)	132 (29.7%)	1	1	
	1–5 year	65 (37.4%)	183 (41.2%)	1.267(0.799–2.010)	0.760 (0.383–1.509)	0.433
	> 5 year	72 (41.4%)	129 (29.1%)	1.991(1.251–3.169)	0.773 (0.361–1.655)	0.508
Income generating work in prison	Yes	28 (16.1%)	157 (35.4%)	1	1	
	No	146 (83.9%)	287 (64.6%)	2.852 (1.821–4.468)	2.539 (1.483–4.347)	0.001*

## Discussion

The finding of this study showed that the magnitude of anxiety among prisoners was 28.2% [*N* = 174 (95%CI, 25–32%)]. This finding was comparable with the studies done in the United Kingdom (27.7%) and Poland (31.8%) [12, 18]. But it was higher than the studies done in Rohtak, Haryana (8%), Chile (8.3%), and India (8.5%) [7, 19, 20] and it was lower than the studies done in Norway (34.6%), Nigeria (77.8%) and Ethiopia (36.1%) [6, 9, 21]. The differences might be due to the sample size, target population, and measurement tool.

The odds of developing anxiety were 4.21 and 3.95 times higher among inmates whose age was ≥48 and 38–47 respectively than those ages 18–27. This could be explained by those older ages are likely to be exposed to a stressful life event and have changes in the brain and nervous system. Also social and family responsibilities may increase the odds of developing anxiety. Those prisoners who lived in urban were 2.48 times more likely to develop anxiety than rural residents. This might be due to the exposure of urban dwellers to different addictive substances and electronic materials like a phone being departed from those things may expose them to anxiety. The odds of developing anxiety were 2.71 times higher among inmates who didn’t do physical exercises than those who did physical exercise. This finding was supported by a study done in the United States of America [22]. This is because exercise makes a lot of neurotransmitters in the brain like dopamine, serotonin, and noradrenaline function the same way as selective serotonin reuptake inhibitors [23]. The odds of developing anxiety were 1.63 times higher among prisoners who had chronic disease than those who didn’t have a chronic disease. This might be due to the chronic disease patients have a long course of the disease and high expenditures for health care which add pressure on them to cause anxiety. Additionally, chronic disease causes an inflammatory reaction that can influence the availability of neurotransmitter precursor amino acids, and these changes are associated with mental health [24]. Inmates who had 1–2 stressful life events were 2.92 than those who hadn’t stressful life events. This is because stress alters the function of glucocorticoid signaling which causes psychopathology [25]. Whereas stressful life events may make inmates more vulnerable to anxiety since they can’t share their matters with their loved ones also they are tangled to solve their and their family problems so it adds burden on them. Those inmates whose duration of punishment was > 5 years were 2.92 times more likely to develop anxiety than those whose punishment duration is < 1 year. This could be due to thinking of the profound loss of liberty and being away from family and loved ones for a long time may cause anxiety. The odds of developing anxiety were 2.54 times higher among prisoners who hadn’t income-generating jobs in prison than those who had income-generating jobs. This might be due to doing the job for income generation may help them fulfill their basic needs and make their life easy and it may divert them from thinking about the prison situation also it may give them hope to continue living.

The limitation of this study was being cross-sectional design, which does not establish a temporal relationship between cause and effect.

## Conclusion

The magnitude of anxiety among prisoners was high. Age ≥ 48 and 38–47, urban residence, not doing physical exercise, having a chronic disease, having 1–2 stressful life events, duration of punishment > 5 years, and lack of income-generating job in prison were significantly associated with anxiety. Giving special attention and being supportive to older age and those who have a chronic disease, facilitating physical exercise, creating job opportunity in the prison, and giving training for prisoners on stress coping mechanism and anxiety is recommended.

## Data Availability

The data used to support the findings of the current study can be obtained from the corresponding author on reasonable request via hanuahi@gmail.com.

## Abbreviations

AOR: Adjusted Odds Ratio
COR: Crude Odds Ratio
ODK: Open Data Kit
WHO: World Health Organization
